# Multiple Serial Casting for Recurrent Clubfoot in Arthrogryposis Corrects Deformity With Diminishing Returns

**DOI:** 10.7759/cureus.54398

**Published:** 2024-02-18

**Authors:** Jeffrey M Henstenburg, Nikki Sutliff, Alexander Rompala, Amanda Stutman, Kyrillos M Akhnoukh, Harold J Van Bosse, Sarah B Nossov

**Affiliations:** 1 Orthopedic Surgery, Rothman Orthopedic Institute, Philadelphia, USA; 2 Orthopedic Surgery, Shriners Children’s Hospital, Philadelphia, USA; 3 Orthopedic Surgery, SUNY Downstate College of Medicine, Brooklyn, USA; 4 Orthopedic Surgery, SSM Health Cardinal Glennon Children's Hospital, Saint Louis, USA

**Keywords:** ponseti, arthrogryposis multiplex congenita, pirani, casting, treatment, clubfoot, arthrogryposis

## Abstract

Purpose: Arthrogryposis multiplex congenita (AMC) consists of more than 400 conditions involving severe joint contractures of at least two or more body regions. Management of clubfoot in patients with AMC is notoriously challenging, with a higher likelihood of recurrence than idiopathic clubfoot, which can be treated using the Ponseti technique to avoid or delay more invasive procedures. The purpose of this study is to determine the utility of multiple serial casting as a treatment of clubfoot in AMC using Pirani scores as an objective measure of deformity.

Methods: Pirani scores were retrospectively collected from 17 AMC patients with a total of 30 clubfeet and two years follow-up from initiation of treatment. Patients with a minimum of three casting series were included. Pre-treatment and post-treatment deformity scores were examined across casting series using analysis of variance (ANOVA) statistical analysis.

Results: The first series pre-treatment Pirani score improved from 4.80±1.54 to 1.68±1.48 (p<0.001). The second series improved from 4.23±1.03 to 2.72±0.916 (p<0.001). The third series had the smallest improvement from 3.87±1.07 to 2.82±1.02 (p<0.001). Change in Pirani scores showed a significant decrease from the first series to the second (p=0.001) and third (p<0.001). In addition, the number of casting days was found to significantly affect the change in scores during the third series (p=0.038).

Conclusions: The Ponseti technique can improve clubfoot in AMC as measured by the Pirani score. Data shows that early intervention yields better results, with a diminished yet effective ability to elicit change over time.

## Introduction

Treatment of idiopathic clubfoot with the Ponseti technique has been demonstrated to yield high success rates compared to early and more aggressive surgical intervention [1]. Compared to idiopathic clubfoot, non-idiopathic clubfoot is usually more severe, stiffer, and often associated with syndromic conditions. Non-idiopathic clubfoot was initially thought to be untreatable by the Ponseti technique; however, subsequent studies have documented the utility of this technique for more complex deformities [2-7]. Patients who have arthrogryposis multiplex congenita (AMC) and clubfoot have non-idiopathic clubfoot.

Non-idiopathic clubfoot tends to be more challenging to treat than idiopathic clubfoot. Patients who have AMC and clubfoot are often bilateral and more rigid than idiopathic clubfoot, with a much higher recurrence rate [4-7]. The indications for the extent of treatment may depend on ambulatory potential, and residual deformity may be acceptable based on the patient's ambulatory status and family goals. The goal of clubfoot treatment for AMC patients is a plantigrade and brace-able foot, functioning as a stable platform for ambulation. In many cases, it may be tempting to surgically intervene earlier than necessary due to the difficulty of achieving and maintaining a successful correction [7].

Past literature advocated for early aggressive surgical intervention for non-idiopathic clubfoot deformity out of concern for the high relapse rate [4-7]. However, studies have shown that the non-idiopathic clubfoot has a high relapse rate regardless of initial treatment [8-10]. In cases where the initial intervention was surgical, additional procedures were often required. Each additional surgical intervention causes increased scar formation, stiffness, and contracture, making treatment after subsequent relapses more difficult. Furthermore, there is no consensus on defining failure versus acceptable relapsed deformity. There is a growing body of literature supporting surgical delay or avoidance during years of maximal childhood growth and instead using serial Ponseti style casting to maximize chances of successful outcomes [4-7,11]. Church et al. specify the age range of 4.0-6.9 years as a period of rapid growth and a target age range to continue serial casting [11].

Relapses of clubfoot in AMC can be treated with casting and Achilles tenotomies as needed. This serves to delay further surgical intervention, as invasive interventions may not be as successful in this group until patients approach skeletal maturity. The goal of treatment is to achieve a pain-free, plantigrade foot. Although some use this technique in practice, there is no supportive literature describing the long-term outcomes of this technique for this population.

Therefore, the purpose of this study was to describe the mid-term outcomes of serial Ponseti-style management of clubfoot in AMC. We use Pirani scores, ambulatory status, and the need for surgical intervention as measures to define improvement and relative success [12].

## Materials and methods

For this retrospective case series, 17 patients with AMC and clubfoot deformities who underwent multiple casting series from 2011 to 2019 were identified from the institution's clubfoot database. Of the 17 AMC patients, there were six cases of amyoplasia, six unspecified, four distal AMC, and one polymicrogyria, perisylvian with cerebellar hypoplasia and arthrogryposis (PMGYCHA) (Table 1). Of these 17 patients, eight had previous surgery at outside institutions consisting of one round of bilateral Achilles tenotomies. One of those eight patients had two additional rounds of bilateral Achilles tenotomies and a right adductor tenotomy.

**Table 1:** Demographics PMGYCHA, polymicrogyria, perisylvian, with cerebellar hypoplasia and arthrogryposis

Patient	Sex	Side affected	Type of AMC	Previous surgery	Age at start (years)	Age at latest follow-up (years)
1	M	L	PMGYCHA	No	3.16	5.96
2	F	L	Amyoplasia	No	0.25	5.23
	-	R	-	-	0.25	5.23
3	M	L	Amyoplasia	B/L Achilles tenotomies - 5 months of age	1.32	4.35
	-	R	-	-	1.32	4.35
4	F	L	Unspecified	B/L Achilles tenotomies - 3 months of age	3.67	5.36
	-	R	-	-	4.67	6.36
5	M	L	Amyoplasia	B/L Achilles tenotomies - 6 months of age	1	2.69
	-	R	-	-	1	2.69
6	M	L	Unspecified	B/L Achilles tenotomies x 3, R adductor tenotomy	2.22	5.11
	-	R	-	-	2.22	5.11
7	F	L	Unspecified	No	0.704	5.66
	-	R	-	-	0.704	5.66
8	F	L	Distal	B/L Achilles tenotomies	1.46	5.42
	-	R	-	-	1.46	5.42
9	M	L	Amyoplasia	No	0.5	5.31
	-	R	-	-	0.5	5.31
10	F	L	Unspecified	No	4.17	8.29
	-	R	-	-	4.17	8.29
11	M	L	Distal	No	5.67	10.09
12	F	L	Unspecified	B/L Achilles tenotomies - 2 months of age	4.58	8.44
	-	R	-	-	4.58	8.44
13	M	L	Distal	No	3.33	7.94
	-	R	-	-	3.33	7.94
14	M	L	Amyoplasia	B/L Achilles Tenotomies - 3 months of age	1.33	6.14
	-	R	-	-	1.33	6.14
15	M	L	Unspecified	No	3.08	4.65
	-	R	-	-	3.08	4.65
16	F	L	Amyoplasia	B/L Achilles Tenotomies	1.92	8.30
17	F	R	Distal	No	2.33	6.80

The study was approved by the institutional review board of our institution. Inclusion criteria required a minimum of two years of follow-up from initiation of treatment, complete Pirani score data, and at least three series of casting treatments, regardless of prior treatment at outside institutions. Patients who had been previously treated at another institution were included. Therefore, first casting refers to the initial series performed at the study institution. Patients were selected from this institution’s pediatric population. Patients were excluded for incomplete measurements or treatment.

**Figure 1:**
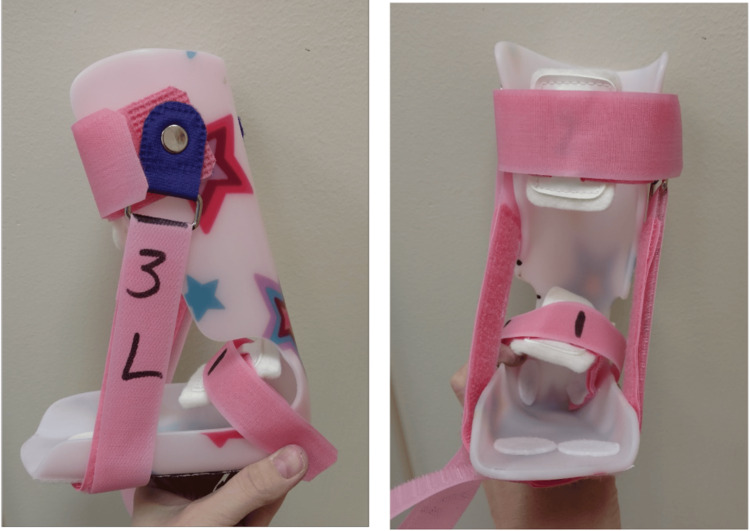
AFOs used post-operatively AFOs, ankle-foot orthoses

Long-leg plaster casts were applied using a Ponseti-style technique. The frequency of cast changes varied from twice a week to once every three to four weeks, depending on the schedule of the family and their distance from the institution. Ponseti casting was usually, but not always, followed by an Achilles tenotomy. An Achilles tenotomy was performed at the surgeon's discretion if they believed greater ankle dorsiflexion was needed at the final cast of a series. A surgical date was chosen based on the estimated number of serial casts needed for correction and not on Pirani scores. Whenever possible, the timing of the casting series was arranged so that Achilles tenotomies could be performed in conjunction with other unrelated procedures (such as hamstring tenotomy and hip flexor tenotomy). We selected a date for surgery and then planned the series of cast applications in reverse order from that date to ensure all casting was completed on time. Post-operatively, patients were cast for a minimum of three weeks until braces were manufactured, or longer, depending on concurrent orthopedic procedures. Patients were fitted with ankle-foot orthoses (AFOs) or knee-ankle-foot orthoses (KAFOs) for walking during the day (Figure 1). These braces were molded prior to placement of the last cast, often in the operating room after the final correction was obtained. At night, subjects used AFOs with leaf-spring ankle and medial and lateral dorsiflexion straps, with the lateral strap more tensioned than the medial to pronate/evert the foot and stretch the Achilles. Bracing was continued until skeletal maturity to prevent or slow progression.

**Figure 2:**
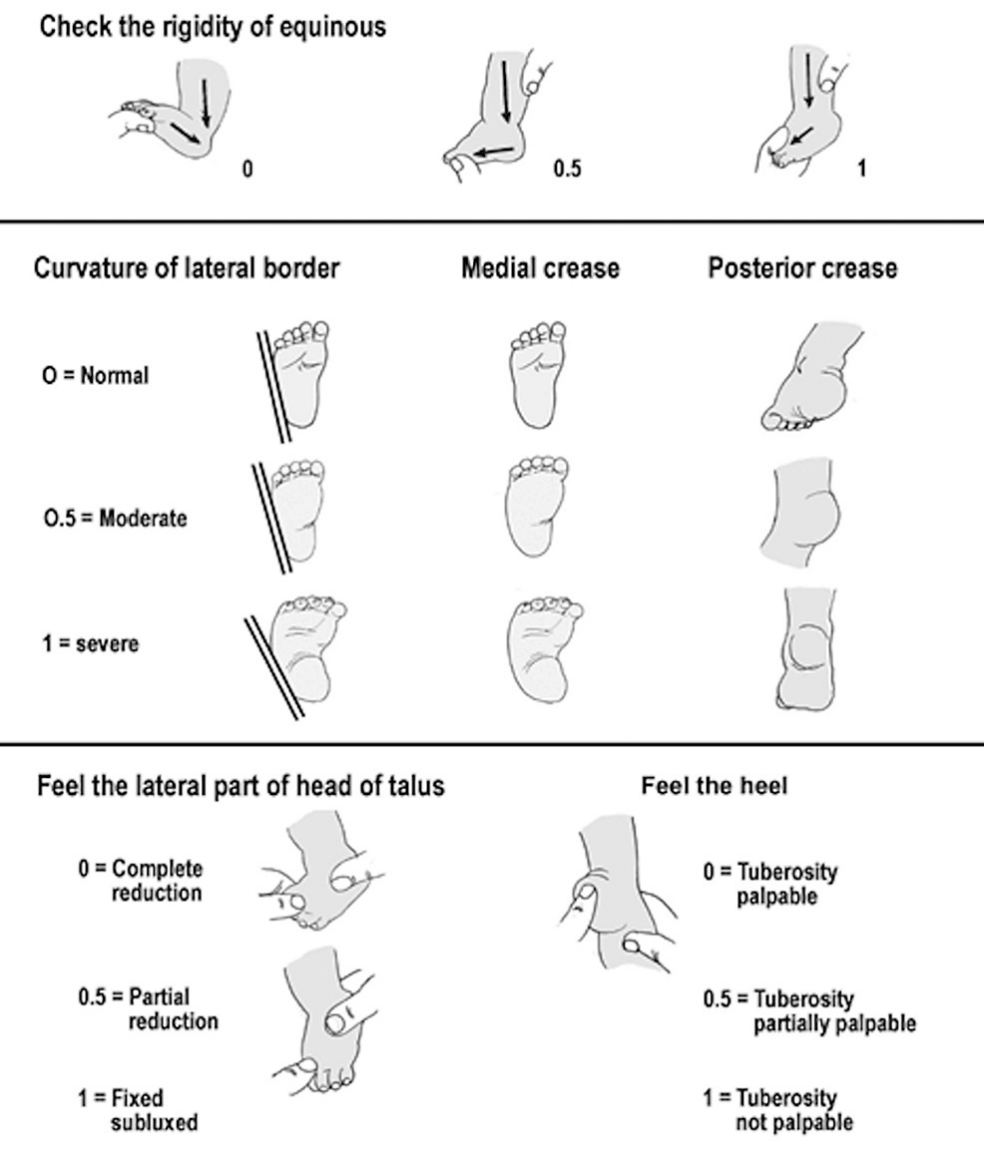
Pirani score chart

Pirani scores were measured by two of the senior authors who are fellowship-trained in pediatric orthopedics (details omitted for double-anonymized peer review) (Figure 2) [13]. "Pre-treatment" measurement refers to the foot appearance at initial presentation to the study site, "pre-series" measurements were those done just prior to starting a cast series, and "post-series" measurements were the first scores recorded after the last cast of a series was removed.

Ordinal composite values for Pirani scores were calculated for initial deformity and the final result after each series. Statistical analysis was performed utilizing repeated measures analysis of variance (ANOVA) with Greenhouse-Geisser correction where appropriate and post-hoc Bonferroni correction for multiple comparisons. In addition, linear regression was used to examine the effects of casting days and the number of casts on improvement in Pirani scores. Statistical analysis was performed using SPSS Statistics Version 25.0 (IBM Corp, Armonk, NY).

## Results

Seventeen patients with a total of 30 treated clubfeet (16 male patients and 14 female patients) met inclusion criteria and were retrospectively reviewed (Table 2). The average age of the cohort was 2.31±1.54 years at the time of first casting at the institution. The average age of the cohort at the final casting measurement of the final casting series was 6.04±1.75 years. The first casting series lasted an average of 59.4±32.5 days (~8 weeks) with 7.13±3.82 casts. The second casting series lasted an average of 49.2±39.6 days (~7 weeks) with 6.67±2.98 casts. Finally, the third series lasted an average of 34.9±18.1 days (~5 weeks) with 5.07±1.68 casts. Achilles tenotomies were conducted on 16 of 17 of the patients included in the study.

**Table 2:** Pirani score, number of casts, and casting days across multiple clubfoot casting series in AMC AMC, arthrogryposis multiplex congenita; SD, standard deviation

	Series 1			Series 2			Series 3		
	Mean	Range	SD	Mean	Range	SD	Mean	Range	SD
Initial Pirani score	4.8	(2.5-6)	1.09	4.2	(2.4-6)	1.03	3.9	(2-6)	1.07
Final Pirani score	1.7	(0-5)	1.49	2.7	(1-4.5)	0.92	2.8	(0.5-5)	1.02
Change in Pirani score	3.1	(-0.5-6)	1.69	1.5	(-0.5-4)	1.06	1.1	(-0.5-3.5)	1.00
Number of casts	7.1	(3-17)	3.82	6.7	(3-14)	2.98	5.1	(2-8)	1.68
Number of days	59.4	(14-133)	32.49	49.2	(14-150)	39.57	34.9	(7-70)	18.13

The mean Pirani score improved by 3.12±1.69 from the start of the first series (pre-treatment, 4.80±1.54) to post-treatment (1.68±1.48) (p<0.001) (Table 3, Figure 2). The mean Pirani score improved by 1.50±1.06 from the start of the second series (4.23±1.03) to post-treatment (2.72±0.916) (p<0.001). The mean Pirani score improved by 1.05±1.00 from the start of the third series (3.87±1.07) to post-treatment (2.82±1.02) (p<0.001). There was also a significant difference between the pre-treatment Pirani scores before the first series and the post-treatment scores at the conclusion of the third series (4.80±1.54 vs. 2.82±1.02, p<0.001) (Table 4). There was less improvement in the second and third series compared to the first (first to second p=0.001; first to third p<0.001). When comparing the changes observed during a casting series, the amount of change obtained during the second series was not different from the amount of change obtained during the third series (p=0.137).

**Table 3:** Differences in Pirani scores between pre-treatment and post-treatment for each series and between initial Pirani score and final Pirani score

	Pirani score difference	SD	95% CI lower	95% CI upper	P-value
Series 1 pre-treatment vs. series 1 post-treatment	3.12	1.69	2.49	3.75	<0.001
Series 2 pre-treatment vs. series 2 post-treatment	1.50	1.06	1.10	1.89	<0.001
Series 3 pre-treatment vs. series 3 post-treatment	1.05	1.00	0.68	1.42	<0.001
Series 1 pre-treatment vs. series 3 post-treatment	1.98	1.30	1.50	2.47	<0.001

**Figure 3:**
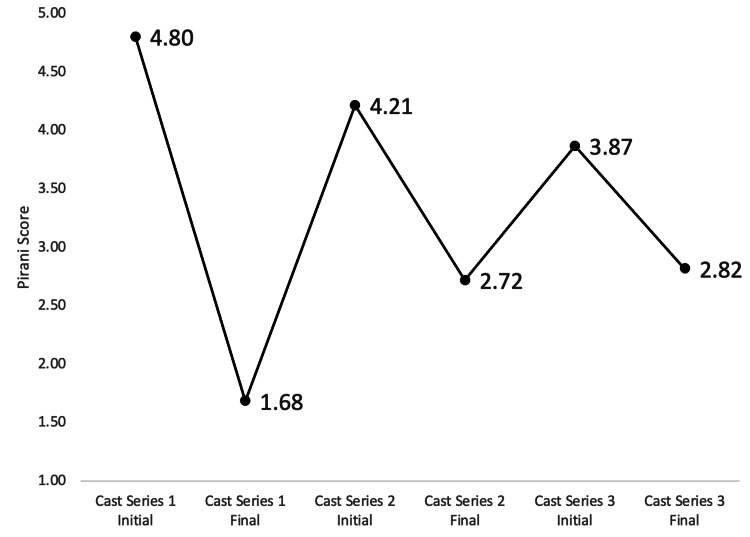
Pirani scores for casting series

**Table 4:** Pairwise comparisons of change in Pirani score for each series *Adjustment for multiple comparisons: Bonferroni

Series		Difference between change in Pirani scores	SD	95% CI lower	95% CI upper	P-value*
1	2	1.620^*^	0.385	0.627	2.613	0.001
	3	2.067^*^	0.370	1.111	3.022	0.000
2	1	-1.620^*^	0.385	-2.613	-0.627	0.001
	3	0.447	0.211	-0.099	0.992	0.137
3	1	-2.067^*^	0.370	-3.022	-1.111	0.000
	2	-0.447	0.211	-0.992	0.099	0.137

In the multivariate analysis, there was no correlation between number of days or number of casts and the improvement of Pirani scores for the first two series (p>0.05). There was also no correlation between the number of casts and the change in Pirani scores for the third series (p>0.05). For the third series of casts, the multivariate analysis was significant (p=0.038) and there was a moderate association (Pearson’s correlation coefficient r=0.499, p=0.0005) between the number of days cast and the improvement in the Pirani scores. The coefficient of determination, r-squared, is 0.249, indicating that the number of days cast is responsible for 24.9% of the variability in Pirani scores; increasing the number of days cast in the third series does improve the Pirani scores, but 75.1% of the random variability still remains.

The average time from the start of the first series to the final cast removal of the third series was 44.3±15.7 months. All 17 subjects had their most recent follow-up within a year, and 14 had their most recent follow-up within six months of data analysis. The average time from the end of the third series until the final follow-up was 27 months for a mean study length of 71.0±13.6 months.

Sixteen of 17 subjects were ambulators at the most recent follow-up. Eight did not require assistance for ambulation, while eight subjects required an assistive device for ambulation (such as a walker), and one subject was confined to a wheelchair with minimal ability to ambulate despite full assistance.

Seven subjects went on to have additional casting series. One subject had three additional casting series, two of which were completed with an Achilles tendon lengthening, and the final casting was followed by a posterior ankle release. This subject did not require assistance for ambulation at the 91-month follow-up. No other subjects went on to have foot surgery beyond Achilles tenotomies to correct their clubfeet during the follow-up period of this study. Twelve patients had other lower extremity procedures to address knee and hip joint contractures not directly related to their clubfoot procedure.

## Discussion

Nearly 25% of all clubfoot deformities are non-idiopathic [14,15]. One study found a cohort of 357 clubfoot patients to include 24% with non-idiopathic etiologies, AMC and myelomeningocele being the most common underlying diagnoses [14]. The high percentage of non-idiopathic clubfoot patients demonstrates the need to define an individualized set of treatment guidelines. Similar to the evolution of the treatment of idiopathic clubfoot, severe deformity in AMC was historically treated with surgical correction; casting was used only to “buy time” [16]. Talectomy has even been recommended as a primary procedure for severe clubfoot in AMC [17,18]. However, long-term follow-up studies documented poor satisfaction with primary talectomies [19]. Others have advocated for early posterior-medial release, but follow-up studies have noted high relapse rates with low long-term success [8,16,20,21]. More recently, authors have reported success in avoiding surgical intervention by serially casting clubfeet in AMC [3,5-7].

Serial Ponseti casting in the treatment of clubfoot in AMC can be successful in improving foot morphology to allow comfortable bracing and stable weight-bearing. Pirani scores showed significant improvement at follow-up compared to pre-treatment; however, the degree of change decreased with each successive casting series. This trend is expected to continue with further casting series. Although the ability to correct clubfeet deformity decreased over time, it is interesting to note that the benefits of Ponseti casting can be successfully applied to older children. This is demonstrated by the fact that 16 of the 17 subjects were ambulatory at their most recent follow-up and had an average age of 6.04 years. One subject required further surgical intervention: a posterior soft tissue ankle release. 

Ponseti casting management for non-idiopathic clubfoot has been shown to approach a 90% success rate in some series, which is consistent with our findings [6,14,22]. A modified technique described by van Bosse et al. includes an optional initial Achilles tenotomy in addition to Ponseti casting, demonstrating a 90% success rate [7]. Other studies show less favorable long-term outcomes with high initial success. Patients demonstrated a 45-50% recurrence rate, leading to surgical intervention in 20-25% of subjects [4,15,23]. When directly comparing early soft tissue release to Ponseti style management, Kowalczyk et al. found that while most subjects eventually had a soft tissue release, there were fewer complications when Ponseti casting was attempted first compared to those that had earlier soft tissue release [5]. This demonstrates that when intervention efforts fail, early Ponseti management efforts may still lead to improved outcomes. Several studies have reported high rates of success with the use of Ponseti casting in idiopathic clubfoot recurrence [24]. While these results cannot be directly applied to the AMC population, it does provide justification for the implementation of Ponseti casting in non-idiopathic clubfoot recurrence.

Few studies have attempted to directly assess patient-reported outcomes following serial Ponseti style management of non-idiopathic clubfoot. Church et al. examined pediatric outcomes scores and gait analysis of clubfeet in AMC compared to idiopathic clubfeet after Ponseti management. Subjects with AMC demonstrated worse outcomes overall compared to idiopathic clubfeet [11]. Unfortunately, patient-reported outcomes scores were unavailable for all subjects included in this study.

There were several limitations to this study. There was a large standard deviation for the number of days included in each cast series. This is likely due to the nature of the institution, where many patients travel long distances for treatment, and the casting schedule is either compressed or stretched out for the convenience of the family. Also, the retrospective study series was small and underpowered to detect the effects of cast number and casting duration on each series. Although an effect of casting days was detected in the third series, it was not very powerful. Given that this series demonstrated the least amount of improvement, it is unclear whether there is a clinical correlation to this association. Additional research with a larger cohort would strengthen the associations elicited in this study while guiding optimal management for patients with clubfoot in AMC.

This study did not account for other aspects affecting treatment success, including talar deformities and failure to follow bracing protocols. Family compliance was not measured and may be difficult to quantify. In the study cohort, 14 of 17 patients (82.4%) continued to present to the clinic at the time of this study, indicating a high rate of follow-up for continued treatment.

The average age of the cohort at the start of the first series was 2.31 years. Either due to the referral pattern and potential need for long-distance travel to the institution or delays in diagnosis, some patients started their initial series of Ponseti cast treatment after infancy. The literature indicates that patients with idiopathic clubfeet who received treatment between one and three months of age yielded lower relapse rates and better clinical outcomes than older age groups [25]. Therefore, many patients in this study may be considered to have a delay in treatment. Of note, the average age at final casting was 6.04 years, demonstrating that Ponseti casting is still beneficial for older children. Additionally, some patients may have been treated at other institutions before receiving care at the study institution.

One subject did not complete serial casting treatment because they went on to have more extensive surgery than an Achilles tenotomy. Not all subjects were mature at the time of their final follow-up and thus may go on to have surgical procedures for clubfoot correction. A longer follow-up is needed to determine lasting success.

Patients who went on to have three or more rounds of serial casting represent the most affected cohort of clubfoot in AMC. The subjects in this study most likely represent the most difficult-to-treat patients and likely do not represent outcomes of patients with less severe forms of clubfoot in AMC.

Finally, patient-reported outcome measures were not included in this study. While objective clinical measurements may have improved after casting, this does not directly translate to improved functional capacity or quality of life.

Despite these limitations, the authors interpret that the data from this study does support the use of serial casting in patients with AMC and clubfoot. A multi-center cohort would be helpful in obtaining larger numbers to elucidate if a particular subset of AMC may ultimately require this sort of treatment, and if success rates may be different with particular syndromic diagnoses in this heterogeneous population.

## Conclusions

Clubfoot in AMC has a very high likelihood of recurrence. Recurrent deformity in this population can be repeatedly improved with the Ponseti technique, as measured by the Pirani score. Earlier treatment yields better outcomes with an effective yet diminished ability to elicit change over time. The concept of accepting residual deformity may be elucidated in future studies by comparing a cohort of patients who receive dramatic interventions using patient satisfaction outcome measures.
